# Against Repurposing Methadone for Glioblastoma Therapy

**DOI:** 10.3390/biom10060917

**Published:** 2020-06-17

**Authors:** Tatjana Vatter, Lukas Klumpp, Katrin Ganser, Nicolai Stransky, Daniel Zips, Franziska Eckert, Stephan M. Huber

**Affiliations:** 1 Department of Radiation Oncology, University of Tübingen, 72076 Tübingen, Germany; Tatjana.Vatter@uni-ulm.de (T.V.); lukas.klumpp@med.uni-tuebingen.de (L.K.); katrin.ganser@med.uni-tuebingen.de (K.G.); nicolai.stransky@med.uni-tuebingen.de (N.S.); daniel.zips@med.uni-tuebingen.de (D.Z.); franziska.eckert@med.uni-tuebingen.de (F.E.); 2 Department of Pharmacology, Toxicology and Clinical Pharmacy, Institute of Pharmacy, University of Tübingen, 72076 Tübingen, Germany; 3 German Cancer Consortium (DKTK), Partner Site Tübingen, Tübingen, Germany, and German Cancer Research Center (DKFZ), 69120 Heidelberg, Germany

**Keywords:** ionizing radiation, flow cytometry, cell cycle regulation, clonogenic survival, colony formation assay, human glioblastoma cell lines, T98G, A172, U87MG, U251

## Abstract

Methadone, which is used as maintenance medication for outpatient treatment of opioid dependence or as an analgesic drug, has been suggested by preclinical in vitro and mouse studies to induce cell death and sensitivity to chemo- or radiotherapy in leukemia, glioblastoma, and carcinoma cells. These data together with episodical public reports on long-term surviving cancer patients who use methadone led to a hype of methadone as an anti-cancer drug in social and public media. However, clinical evidence for a tumoricidal effect of methadone is missing and prospective clinical trials, except in colorectal cancer, are not envisaged because of the limited preclinical data available. The present article reviews the pharmacokinetics, potential molecular targets, as well as the evidence for a tumoricidal effect of methadone in view of the therapeutically achievable doses in the brain. Moreover, it provides original in vitro data showing that methadone at clinically relevant concentrations fails to impair clonogenicity or radioresistance of glioblastoma cells.

## 1. Introduction

Methadone is a synthetic opioid with an asymmetric carbon atom giving rise to two enantiomeric forms: R(−)-(L-)methadone (levomethadone) and S(+)-(D-)methadone (dextromethadone). It was developed in 1937 under the name Polamidon (Hoechst 10820) [1] by Gustav Ehrhart and Max Bockmühl, both German chemists at the IG Farben company [2]. The analgesic effect of methadone was discovered in 1942, and in 1945, methadone was finally approved as an analgesic drug in Germany [3]. After World War II, the US American company Eli Lilly acquired the rights for Polamidon and distributed the substance under the new name methadone [4]. In 1964, studies at the Rockefeller Hospital demonstrated that methadone is effective in maintenance treatment for long-term heroin addiction by “relieving the narcotic hunger and inducing sufficient tolerance to block the euphoric effect of an average illegal dose of diacetylmorphine” [5]. In the clinical setting and in laboratories, the racemic mixture of methadone usually is applied. R(−)-(L-)methadone has analgesic activity, and S(+)-(D-)methadone is discussed to be less active [6] or to have antagonistic activity to the *N*-methyl-d-aspartate (NMDA) receptor affecting morphine tolerance [7].

Glioblastoma multiforme represents the most common primary brain tumor in adults, with approximately 4 cases per 100,000 people per year [8]. Current standard therapy comprises 5-aminolevulinic acid fluorescence- and/or intraoperative MRI-guided surgical resection, followed by fractionated radiotherapy (60 Gy in 30 fractions) with concomitant temozolomide chemotherapy and subsequent temozolomide maintenance therapy for patients suitable for this treatment regimen [9]. The latter may be combined with Tumor-Treating Fields electrotherapy [10]. Glioblastoma cells may be highly radioresistant and, depending on their o-6-methylguanine-DNA methyltransferase activity, also temozolomide resistant. Moreover, glioblastoma cells diffusely infiltrate the brain tissue, forming secondary foci distance from the primary lesion. Glioblastoma cells that already have spread into the healthy brain parenchyma prior to therapy cannot be eliminated by macroscopic complete resection of the tumor and radiotherapy. The latter usually targets the tumor bed with approximately 20 mm safety margin plus (potentially infiltrated) adjacent edematous regions. This infiltrative behavior in concert with a high therapy resistance of the tumor most probably underlies the limited prognosis of glioblastoma patients with a median overall survival of below two years and age-dependent five-year survival rates of 5–19% [8,10,11].

Thus, better therapy concepts are urgently needed and new strategies, such as immunotherapy, oncolytic virus therapy or electrotherapy, but also repurposing approved drugs for anti-glioblastoma therapy, might be an option. Conceptually, drugs with anti-glioblastoma activity may be identified by a retrospective analysis of superior survival of patients with comorbidity-dependent drug prescription. Such a scenario has been initially proposed for the anti-epileptic drug valproic acid [12]. In addition, evidence from hypothesis-driven preclinical experiments might also hint to druggable targets in oncogenic pathways. In the case of methadone, in vitro data from the early 1990s have suggested that autocrine opioid signaling impairs lung cancerogenesis in a negative feedback loop, which seems to be interrupted in smokers by nicotine [13,14,15]. Later on, preclinical data of a group in Ulm, Germany, and others suggested anti-tumoral activity of methadone in leukemia and also in glioblastoma cell lines [16,17,18,19]. The latter finding, once overhyped by the public media, stirred up hope for an efficient anti-glioblastoma therapy. As a result, some glioblastoma patients and their families started seeking ways to get prescriptions for methadone on an individualized basis in hope for a more positive course of the disease. German medical societies such as the DGHO (German Society of Hematology and Oncology [20]) and research funding organizations such as the Deutsche Krebshilfe (German Cancer Aid [21]) published statements stressing the missing evidence for the use of methadone as an anti-cancer drug.

As discussed below, beyond addiction, methadone may exert severe side effects. Moreover, therapeutic and toxic windows of methadone concentration seem to be narrow, justifying its prescription for anti-glioma therapy only upon proof of its efficacy. The present article introduces potential molecular methadone targets that are expressed by glioblastoma cells. In addition, this article summarizes reported preclinical and clinical evidence for a tumoricidal action of methadone and presents our own original data on the effect of methadone on clonogenic survival and radioresistance of glioblastoma cells in vitro. To begin with, the next paragraphs give an overview about the pharmacokinetics of methadone, with an emphasis on therapeutically relevant methadone concentrations.

## 2. Methadone Pharmacokinetics

One hallmark of the pharmacokinetics of methadone is its large interindividual heterogeneity (for reviews, see [22,23]). The bioavailability ranges from 41% [24] up to 100% [25]. Reported half-life varies between 4.2 and 130 h [26]. Plasma protein binding is about 80–90% [27], with most methadone bound to α_1_-globulins. A study in rats showed that after a withdrawal of opioids, levels of α_1_-globulins increased and the concentration of free methadone decreased [28], indicating a need for increased doses. On top of that, cancer patients were found to have higher levels of α_1_-globulins, and hence, lower concentrations of unbound methadone [29]. Body clearance differed in one study from 0.06 up to 0.34 L/min [24], whilst others found a remarkable variation of 0.023 up to 2.1 L/min in cancer patients, maybe due to drug–drug interactions [26].

One potential reason for these interactions is the extensive metabolism of methadone by several cytochrome P450 enzymes (for a review, see [30]); the major contributor to methadone metabolism is CYP3A4, and CYP2D6 plays a minor role. Several other CYP enzymes are also involved, such as CYP2B6, CYP2C8 and CYP2C9, some of which are implicated in contributing to the heterogeneity by polymorphisms but also by drug–drug interactions. Elimination of both methadone and its metabolites occurs via renal and fecal elimination, whereas the proportion, again, varies from person to person, even though renal elimination seems to be responsible to a larger extent more frequently [31]. Table 1 summarizes clinically or clinicopathologically observed methadone concentrations in blood, brain or cerebrospinal fluid.

The only clinical examination of methadone in one glioma patient so far measured a basal plasma concentration of 182 ng/mL (0.59 µM) after an oral uptake of 30 mg methadone [19]. In general, effective doses for its main use, opioid addiction, are 60–120 mg daily [32], with recommended plasma concentrations in the range of 150–600 ng/mL (0.5–2 µM) [33]. Concentrations above 700 ng/mL (2.3 µM) are assumed to be associated with toxic effects [34]; however, the median concentration of methadone in methadone-related deaths was only 500 ng/mL (1.6 µM) [35].

Regardless of the exact interpretation of these data, it is safe to assume that there is also profound variability in tolerability to methadone, especially contemplating the use in the patient population of glioma patients, differing significantly from drug addict patients. This danger is well depicted in the disproportionally high ratio of methadone in opioid pain reliever-associated deaths in the USA from 1999 to 2010 [36]. Of specific interest for the in vitro testing for an anti-glioblastoma activity are methadone concentrations in brain tissue, which were reported to be between 219 and 2652 ng/mL (0.7–8.6 µM) in methadone-related deaths [37], whereas other authors found even higher concentrations of 3700 ng/mL (12 µM) [38]. These data support the notion that many in vitro experiments—including parts of our work (see below)—use methadone concentrations, which are either clinically irrelevant or could lead to serious adverse events, such as respiratory depression [39,40], but also prolongation of the QTc interval, and torsade de pointes arrhythmias [41,42,43,44]. Whether a partially broken blood–brain barrier in GBM [45] will aggravate or alleviate this problem remains an unanswered question.

General knowledge about the blood–brain barrier permeability of methadone is mainly based on studies in rats, showing an uptake into the brain of 42% of the methadone injected into the common carotid artery [49,50]. Several other groups reported the importance of the efflux transporter p-glycoprotein (MDR1) for the blood–brain barrier permeability of methadone in mice [51,52,53]. Whether polymorphisms in MDR1 may also contribute to different dosing requirements in humans is debated [54,55,56] (for a review of the pharmacogenetics of methadone in general, see [40]).

To conclude, methadone is a highly challenging drug for each clinician, probably necessitating increasing doses slowly, tapering at the end of the treatment, thorough evaluation of potential drug–drug interactions, regular drug concentration measurements and close monitoring of potential adverse effects. Beyond the above mentioned severe toxicities, methadone may diminish quality of life, starting from obstipation and nausea [19], and ending up with sedation, delirium and severe withdrawal symptoms [57]. These real challenges and accompanying risks should be balanced against the potential benefits [58]. The next paragraphs introduce putative molecular targets of methadone in glioblastoma.

## 3. Methadone Target Molecules

Methadone is known as a fully synthetic agonist of the µ-opioid receptor (encoded by the ORPM1 gene) with a 10-fold higher affinity of the R(−)-(L-)- (EC_50_ = 10 nM) over the S(+)-(D-)-enantiomer (EC_50_ = 100 nM) [59,60]. The µ-opioid receptor can form heteromers with the galanin receptor 1 (GalR1) [61], which dramatically lowers the affinity of the µ-opioid receptor to methadone. At the heteromeric µ-opioid–Gal1-receptor complex, methadone stimulates dramatically lower dopaminergic effects in rat brains as compared to morphine (EC_50_: ~10^−4^ M vs. ~10^−7^ M) [62]. Beyond the µ-opioid receptor, methadone has been shown to directly modulate further target proteins, as summarized in Table 2.

In addition, methadone reportedly inhibits L- and T-type Ca^2+^ channels, with estimated IC_50_ values above 10 µM. Interestingly, it seems that there are different ways of how methadone interacts with the two subfamilies of voltage-gated Ca^2+^ channels. The µ-opioid receptor antagonist naloxone partially reverses the inhibition of the T-type Ca^2+^ channels, which suggests a µ-opioid receptor-dependent inhibition, but not the blockage of L-type Ca^2+^ channels [66].

Furthermore, methadone was also found to inhibit rat α_3_β_4_ nicotinic receptors non-competitively and human M3 muscarinic acetylcholine receptors (AChR) competitively in an enantiomer unspecific manner, with an IC_50_ of 1.9 µM and a K_i_ of 1 µM, respectively [67,75]. R-(+)-2-ethyl-1,5-dimethyl-3,3-diphenylpyrrolinium perchlorate (EDDP), the major metabolite of methadone, has been shown to be an even more potent inhibitor of the rat α_3_β_4_ nicotinic AChR than methadone with an IC_50_ of 0.5 µM [67].

The observation that high doses of methadone may prolong the QTc interval in patients [69] suggests that methadone might act on the human-ether-à-gogo related (hERG1) voltage-gated K^+^ channel, which is crucial for repolarization and duration of the cardiac action potential [76]. As a matter of fact, methadone has been demonstrated to inhibit hERG1 in a µ-opioid receptor-independent manner, with reported IC_50_ values of 2–10 µM [69,77] and a higher potency of S(+)-(D-)-methadone (IC_50_ = 2 µM) over R(−)-(L-)-methadone (IC_50_ = 7 µM) [69]. Furthermore, a direct inhibition of GIRK1/GIRK2 channels by methadone with an IC_50_ of ~53 µM has been found [70].

**Figure 1:**
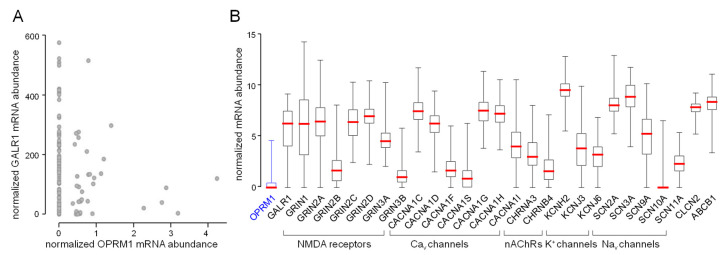
Expression of methadone target molecules in glioblastoma (Illumina HiSeq_RNA Seq V2 glioblastoma dataset (n = 172) of The Cancer Genome Atlas (TCGA))**.** (**A**) Majority of glioblastoma specimens exhibit non-detectable low abundances of the µ-opioid receptor (OPRM1) mRNA. Here, the µ-opioid receptor mRNA abundances are plotted against those of the GALR1 (galanin receptor 1), which forms heteromeric receptors with OPRM1 (values of individual tumors are shown). (**B**) Box whisker plots depicting relative mRNA abundances (TCGA-normalized values) of proposed methadone targets in glioblastoma specimens (medians are highlighted by red lines). Ca_v_ channels: voltage-gated L- and T-type Ca^2+^ channels, nAChRs: nicotinic acetylcholine receptors, NMDA receptors: *N*-methyl-d-aspartate receptors, Na_v_ channels: voltage-gated Na^+^ channels, KCNH2: hERG1 K^+^ channel, CLCN2: ClC-2 Cl^−^ channel, ABCB1: p-glycoprotein (MDR1).

Beside the action of methadone on voltage-gated K^+^ channels, a direct blockage of voltage-gated (Na_v_) Na^+^ channels (Na_v_1.2, Na_v_1.3, Na_v_1.7, and Na_v_1.8) has been demonstrated in mouse peripheral nerves, with IC_50_ values ranging from 86 to 119 µM [71]. Considerably lower, and thus, in the therapeutically achievable plasma concentration range, is the reported methadone IC_50_ value (100 nM) for the µ-opioid receptor-independent blockage of human Cl^-^ channel-2 (hClC2) in T84 intestinal epithelial cells or when stably expressed in HEK293EBNA cells [72]. Finally, methadone has been demonstrated to inhibit human p-glycoprotein (MDR1): methadone was found to block calcein efflux in p-glycoprotein-transfected HEK293 cells with an estimated IC_50_ value above 25 µM and paclitaxel uptake in preparations of human placental inside-out vesicles with a K_i_ of 18 µM [73,74]. To which extent methadone may exert tumoricidal activity via stimulation of the µ-opioid receptor and/or modulation of further target molecules (Table 2 and Figure 1B) is discussed in the next paragraphs.

## 4. Evidence for a Tumoricidal Activity of Methadone

The postulated ability of methadone to enhance cancer therapy [16,17,18] has received considerable attention in recent years, but remains highly controversial. Preclinical evidence for a tumoricidal methadone effect has been obtained in vitro and in mouse models for various tumor identities, such as leukemia [17,18,78,79,80], glioblastoma [16,81,82], neuroblastoma [83], and carcinoma cells [13,14,83,84,85,86]. These studies are summarized in Table 3. In particular, among the tested carcinoma cells, only small cell lung cancer (SCLC) and non-small cell lung cancer cells (NSCLC) show high sensitivity to methadone (IC_50_ in the nanomolar range) in vitro and in ectopic xenograft mouse models [14]. Much lower or undetectable impairment of viability or induction of cell death by methadone (IC_50_ >> 10 µM) has been reported for pancreatic and colorectal adenocarcinoma [86], head and neck squamous cell carcinoma (HNSCC) [84,87], or bladder carcinoma cells [85]. In these reports, co-treatment of methadone resulted in sensitization to cisplatin (bladder carcinoma and HNSCC cells [84,85]), doxorubicin, 5-fluoruracil, paclitaxel (HNSCC cells [84]) or 5-aminolevulinic acid-based photodynamic (ALA-PDT [88]) therapy (HNSCC cells [87]) with similar low potency (IC_50_ ≥ 32 µM) as compared to the effect of the methadone monotherapy in these cells.

Moreover, methadone reportedly decreases proliferation and increases apoptotic cell death of CEM and HL-60 leukemia cells with an IC_50_ of ~15–100 µM. The pro-apoptotic effect of methadone is probably mediated by upregulation of caspase activities and downregulation of anti-apoptotic proteins such as Bcl-xL, XIAP, Bcl-2 or p21 [17,78]. In addition, the cytotoxic effect of methadone in combination with cytostatic agents was tested in primary acute lymphoblastic leukemia (ALL) cells in vitro [18,79,80] and in an ectopic mouse model (20 mg/(kg·BW·d) methadone) [18]. In these studies, methadone (IC_50_ <0.3–≥32 µM) enhanced the viability-attenuating (or cell death-promoting) effect of Bcl-2 targeting by ABT-737 [79], doxorubicin chemotherapy [18], and asparaginase therapy [80]. The doxorubicin-sensitizing action of methadone (20–120 mg/(kg·BW·d) in increasing doses) was also observed in mice ectopically xenografted with ALL cells [18]. Mechanistically, methadone induced the uptake and inhibited efflux of doxorubicin into/from the ALL cells. Doxorubicin, on the other hand, stimulated µ-opioid receptor upregulation [18]. Moreover, a genome-wide shRNA library-based screening identified the µ-opioid receptor as an asparaginase resistance gene [80]. In summary, these preclinical data suggest that clinically achievable concentrations of methadone may impair viability of SCLC and NSCLC lung cancer cells in monotherapy and may sensitize leukemia cells to, e.g., doxorubicin chemotherapy.

Doxorubicin only poorly penetrates the blood–brain barrier (BBB). Few clinical data from glioblastoma patients are available, e.g. after i.v. application of BBB-permeant pegylated liposomal doxorubicin [89,90]. These data do not prove high tumoricidal efficacy of this DNA-intercalating drug in glioblastoma, suggesting doxorubicin is not the best choice for second line chemotherapy in patients with recurrent glioblastoma or elevated temozolomide toxicity. Accordingly, doxorubicin is not applied routinely in glioblastoma. Nevertheless, the doxorubicin-sensitizing effect of methadone (3.2-32 µM) was studied in vitro in human A172 and U118MG glioblastoma cells, indicating indeed a doxorubicin-sensitizing action of methadone with IC_50_ in the range of ≤3–~10 µM [16,84]. Additional experiments in A172 cells showed a cisplatin-, 5-fluoruracil- and paclitaxel-sensitizing action of methadone with IC_50_ of about 4 µM, >>32 µM, and >>32 µM, respectively [84].

Methadone alone impaired viability (or induced cell death) in A172 [16,87] and U118MG cells [16] or—in further studies—in U251, U87MG and primary human glioblastoma cells [81,91] as well as in neuroblastoma cells [83] in vitro with very poor efficacies (IC_50_ in the range of ~10 µM–>> 145 µM). Conflictingly, Shi et al., who could not demonstrate a significant apoptosis induction (as defined by annexin-V binding in flow cytometry) with 32 µM (10 µg/mL) methadone in a previous study [87], more recently reported an induction of annexin-V binding in A172 cells by 0.065 µM methadone [82]. Additionally, they showed that methadone (32 µM in [87] and 0.065 µM in [82]) enhances the efficacy of ALA-PDT in A172 cells by upregulation of phosphorylated JNK and Bcl-2 [82,87].

Combined, except the most recent findings from Shi et al. [82], these studies suggest very low anti-glioblastoma efficacy of methadone monotherapy. Accordingly, methadone-mediated growth delay of U87MG tumors ectopically xenografted into mice could be observed at very high doses (up to 240 mg/(kg·BW·d) p.o.), greatly exceeding commonly given doses for opioid addiction therapy, which are in the range around 1–2 mg/(kg·BW·d). In contrast to monotherapy, in vitro studies indicate that methadone at clinically achievable concentrations may enhance the efficacy of experimental therapies [16,82,84]. To analyze whether methadone may also enhance the efficacy of the standard anti-glioblastoma therapy [9], U87MG, U251 and primary human glioblastoma cells were treated with a combination of methadone (1–145 µM) and/or temozolomide (100–200 µM) and/or radiation (4 Gy), and cell survival/death was analyzed by crystal violet staining, annexin-V binding, dehydrogenase activity or ATP measurement. As a result, clinically achievable methadone concentrations are not able to sensitize to temozolomide (apparent methadone IC_50_ >>30–>>145 µM) [81,91], to radiation (apparent methadone IC_50_ >>30µM) or to concomitant temozolomide-radiation therapy (apparent methadone IC_50_ >>30 µM) [91].

Likewise, retrospective clinical data (e.g., [19,92,93,94]) that hint at a tumoricidal activity of methadone in glioblastoma are nonexistent and prospective clinical trials are not envisaged. For metastatic colorectal cancer, in contrast, the MEFOX-trial is being initiated: a phase 1 followed by a randomized phase 2 trial comparing mFOLFOX6 chemotherapy alone with mFOLFOX6 chemotherapy plus methadone (AIO-KRK-0119, EudraCT-No: 2019-004158-26).

To conclude, preclinical data suggest that methadone might be effective as monotherapy in lung cancer or might sensitize to established therapies in certain types of cancer, such as pediatric ALL. In glioblastoma, however, evidence for a tumoricidal and standard therapy-sensitizing action of methadone in clinically achievable concentrations is very weak and missing, respectively. Moreover, the preclinical studies on glioblastoma mainly used methadone-induced cell death or impairment of total dehydrogenase activity (measure of cell viability) as endpoints (see Table 3). From our radio-oncological point of view, it is not so decisive whether a given anti-cancer treatment kills a lower or higher percentage of tumor cells or delays tumor cell proliferation more or less. A much more meaningful measure of therapy efficacy is how many tumor cells maintain their clonogenicity and are capable to regrow the tumor after the end of therapy. Clonogenic survival of tumor cells results in tumor relapse and therapy failure. We, therefore, performed further in vitro experiments that addressed the effect of “supratherapeutic” and clinically relevant concentrations of methadone alone and in combination with ionizing radiation on clonogenic survival in different human glioblastoma cell lines. Since our previous work suggests that blockage of putative methadone targets may decrease clonogenic survival of irradiated tumor cells by impairment of cell cycle arrest [95], we also tested the effect of methadone and ionizing radiation on cell cycle regulation. Prior to that, we analyzed the expression of putative methadone target molecules in glioblastoma, as described in the following paragraphs.

## 5. Methadone in “Supratherapeutic” Concentrations May Modify Cell Cycle but Fails to Impair Clonogenic Survival or Radioresistance of Human Glioblastoma Cells in Clinically Relevant Concentrations In Vitro

Querying the glioblastoma database of The Cancer Genome Atlas (TCGA) for the mRNA abundances of putative methadone targets indicates undetectably low OPRM1 (µ-opioid receptor) mRNA abundance in the majority of glioblastoma specimens (Figure 1A,B, first box plot). In contrast to OPRM1 mRNA, GalR1 mRNA encoding for the galanin receptor 1 is abundant in most glioblastoma specimens (Figure 1A,B, second box plot). This might suggest that the µ-opioid receptors in glioblastoma predominately form heteromeric complexes with the galanin receptor 1 that exhibit low methadone affinity (see above). Figure 1B shows the mRNA abundances of further putative methadone targets in glioblastoma specimens.

**Figure 2:**
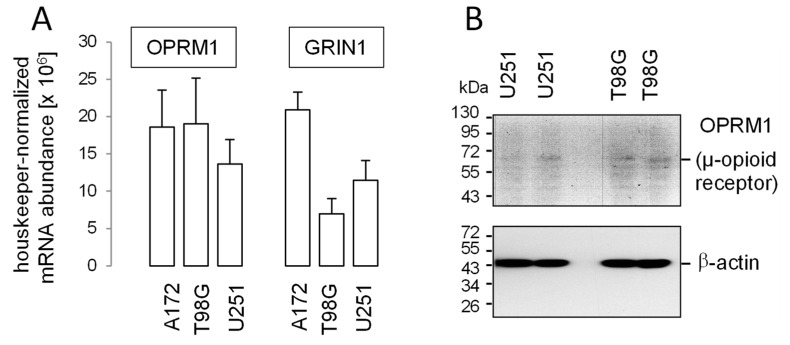
Abundance of µ-opioid (OPRM1) and *N*-methyl-d-aspartate (NMDA) receptors (GRIN1) in human glioblastoma cell lines. (**A**) Mean (SE, n = 3) housekeeper-normalized abundances of OPRM1 (**left**) and GRIN1 (**right**) mRNA in A172, T98G, and U251 cells. (**B**) Immunoblots from total cell lysates of U251 (left) and T98G (**right**) cells separated by SDS-PAGE and probed against OPRM1 (μ-opioid receptor, upper blot) and for loading control against β-actin (lower blot) Human glioblastoma cell lines T98G, A172 and U87MG (see below) were obtained from the American Type Culture Collection (ATCC, Bethesda, MD, USA) and grown in 10% fetal bovine serum (FBS)-supplemented RPMI-1640 medium. The U251 cells were a kind gift from Dr. Luiz O. Penalva (Graduate School of Biomedical Sciences, UT Health San Antonio, Texas) and grown in Dulbecco’s modified Eagle’s medium containing 4500 mg glucose/l and 10% FBS. Total RNA was isolated using the NucleoSpin RNA Kit (Macherey-Nagel, Düren, Germany). OPRM1-, GRIN1- and housekeeper β-actin (ACTB)-, pyruvate dehydrogenase beta (PDHB)-, and glyceraldehyde-3-phosphate dehydrogenase (GAPDH)-specific fragments were amplified by the use of SYBR Green-based quantitative real-time PCR (QuantiTect Primer Assay QT00001512, QT00082089, QT00095431, QT00031227, and QT01192646, QIAGEN, Venlo, Netherlands, and 1Step RT qPCR Green ROX L Kit, highQu, Kraichtal, Germany) in a Roche LightCycler^®^ 480 (Roche, Mannheim, Germany). Abundances of the individual mRNAs were normalized to the geometrical mean of the three housekeeper mRNAs. For western blotting, cells were lysed in a buffer (containing in mM: 50 N-2-hydroxyethylpiperazine-N-2-ethanesulfonic acid (HEPES) pH 7.5, 150 NaCl, 1 ethylenediaminetetraacetic acid (EDTA), 10 sodium pyrophosphate, 10 NaF, 2 Na_3_VO_4_, 1 phenylmethylsulfonylfluoride (PMSF) additionally containing 1% triton X-100, 5 µg/mL aprotinin, 5 µg/mL leupeptin, and 3 µg/mL pepstatin) and separated by SDS-PAGE under reducing condition. Blots were blocked in tris(hydroxymethyl)aminomethane-buffered saline (TBS) containing 0.05% Tween 20 and 5% non-fat dry milk for 1 h at room temperature. The membrane was incubated overnight at 4 °C and for 1 h at room temperature with recombinant anti-µ-opioid receptor antibody [UMB3] [96] (ab227067, Abcam, Berlin, Germany, 1:500) and anti-β-actin (1:20,000, clone AC-74, #A2228, Sigma-Aldrich, Deisenhofen, Germany), respectively. Antibody binding was detected with a horseradish peroxidase-linked goat anti-rabbit IgG antibody or anti-mouse IgG antibody (Cell Signaling # 7074 and # 7076, respectively; 1:1000–1:2000) incubated for 1 h at room temperature (all antibody dilutions in TBS-Tween/5% milk) and enhanced chemiluminescence (ECL western blotting analysis system, GE Healthcare/Amersham-Biosciences, Freiburg, Germany).

To test for tumoricidal effects of methadone in glioblastoma, we first analyzed expression of the µ-opioid receptor (OPRM1) and the NMDA receptor subunit ζ1 (GRIN1) in our human glioblastoma cell lines A172, T98G and U251 by RT-PCR and in part, by western blotting. As shown in Figure 2A, OPRM1- and GRIN1-specific mRNA could be detected in all three glioblastoma lines. In addition, weak immunoreactive bands at the expected migration range of 47–65 kDa were apparent in blots of T98G and U251 total lysates probed against the µ-opioid receptor (Figure 2B). Together, this suggests expression of µ-opioid receptors in T98G, A172, and U251 glioblastoma cells. Protein expression of µ-opioid receptors has been reported previously for A172 [16,82] and U251 [81], as well as for U87MG [81] human glioblastoma cells (U87MG cells were also used in the present study; see below).

Beyond µ-opioid and ζ1-NMDA (GRIN1) receptors (Figure 2A), expression of further putative molecular methadone targets, such as L-type and T-type voltage-gated Ca^2+^ channels (see Table 2), has been reported previously by our group for U251 and T98G cells [97]. In addition, the glioblastoma dataset of the TCGA (see Figure 1) suggests expression of further methadone target molecules (see Table 2), such as the cardiac voltage-gated K^+^ channels hERG1 (KCNH2) or nicotinic acetylcholine receptors CHRNA3/B4.

Reportedly, electrosignaling and interdependent Ca^2+^ signaling contribute to the stress response of cancer cells by regulating cell cycle progression and promoting their clonogenic survival (forreview, see [98]). Since methadone has been proposed to target hERG1 K^+^ channels and ionotropic receptors (see Table 2) and since hERG1 reportedly [95] and NMDA- as well as nicotinic acetylcholine receptors most probably interfere with electrosignaling, we studied the effect of methadone on cell cycle progression in control and irradiated T98G, A172 and U251 cells by flow cytometry. In particular, we monitored the effect of a “supratherapeutic” methadone concentration (20 µM) in order to modulate the ionotropic nicotinic α_3_β_4_ acetylcholine and NMDA receptors, as well as hERG1 and L- and T-type Ca^2+^ channels (IC_50_s in Table 2) in addition to the µ-opioid receptor.

**Figure 3:**
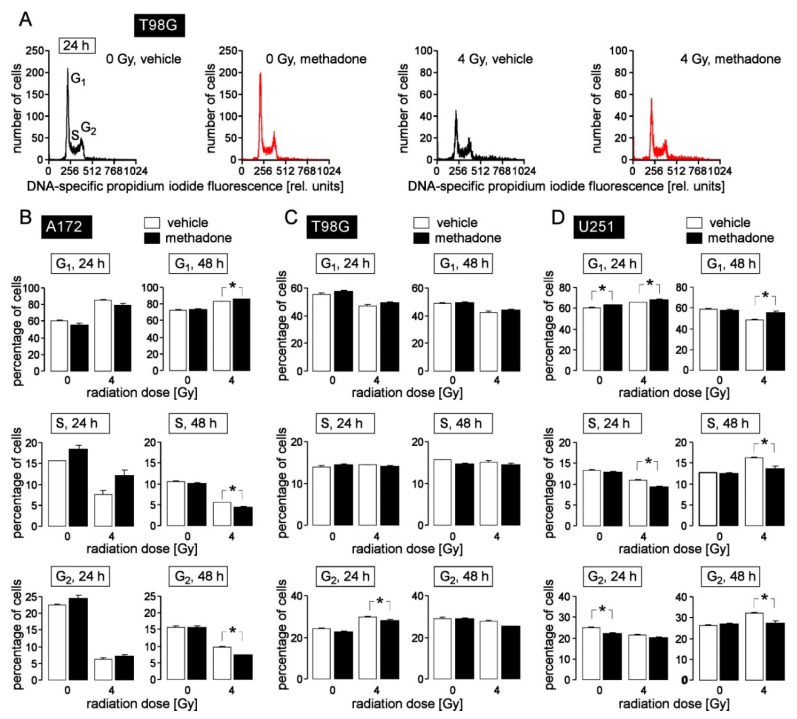
High-dose methadone (20 µM) may modify cell cycle progression in human glioblastoma cells. (**A**) Histograms of permeabilized, propidium iodide-stained T98G glioblastoma cells recorded by flow cytometry (Nicoletti protocol) 24 h after irradiation with 0 Gy (**left**) or 4 Gy (**right**). Cells were pre- (1 h) and co-incubated (24 h) either with 0 μM (vehicle, black) or 20 μM (red) methadone. (**B**–**D**) Mean (± SE, n = 6) percentage of A172 (**B**), T98G (**C**) and U251 (**D**) glioblastoma cells that reside in G_1_ (upper row), S (middle row) and G_2_ (lower row) phase of cell cycle 24 h (**left**) and 48 h (**right**) after irradiation with 0 or 4 Gy. Cells were pre-incubated (1 h), irradiated and post-incubated (24 or 48 h) in the presence of 0 μM (vehicle, open bars) or 20 μM (closed bars) methadone. * indicates *z*p ≤ 0.05, two-tailed (Welch-corrected) t-test with Bonferroni correction for *z* = 16 pairwise comparisons. Cells were irradiated (6 MV photons, single dose of 0 or 4 Gy) using a linear accelerator (LINAC SL25 Philips) at a dose rate of 4 Gy/min at room temperature and post-incubated for further 24 h and 48 h in the absence (vehicle, ethanol) or presence of methadone (20 µM). For cell cycle analysis, cells were permeabilized and stained (30 min at room temperature) with propidium iodide solution (containing 0.1% Na-citrate, 0.1% triton X-100, 10 µg/mL propidium iodide in phosphate-buffered saline, PBS), and the DNA amount was analyzed by flow cytometry (FACS Calibur, Becton Dickinson, Heidelberg, Germany, 488 nm excitation wavelength) in fluorescence channel FL-3 (linear scale, >670 nm emission wavelength). Data were analyzed with the FCS Express 3 software (De Novo Software, Los Angeles, CA, USA).

For cell cycle analysis in flow cytometry, the cellular DNA content was visualized by propidium iodide staining of permeabilized cells, 24 and 48 h after irradiation with 0 and 4 Gy (Figure 3A). As endpoints, the percentage of cells residing in G_1_, S, and G_2_ phase of cell cycle was calculated. Ionizing radiation (4 Gy) induced an increase in G_1_ population and a decrease in S and G_2_ population at 24 and 48 h after radiation in A172 cells (Figure 3B, open bars), suggestive of a radiation-induced G_1_ arrest. In T98G cells, in contrast, radiation (4Gy) stimulated a transient G_2_/M cell cycle arrest that became evident 24 h after irradiation (Figure 3C, open bars) and in U251 cells, a transient G_1_ arrest after 24 h followed by an S and G_2_/M arrest 48 h after irradiation (Figure 3D, open bars).

Methadone (20 µM) when applied concomitantly to radiation and during the 24 and 48 h post-incubation period exerted only little effect on cell cycle progression (compare open and closed bars in Figure 3B–D). In general, methadone delayed G_1_/S transition leading to reduced S and/or G_2_ populations in irradiated A172 (48 h values), T98G (24 h values), and U251 cells (24 and 48 h values) as well as in unirradiated U251 cells (24 h values). This suggests that “supratherapeutic” methadone concentrations may augment radiogenic G_1_ arrest in A172 and U251 cells and delay radiogenic G_2_/M cell cycle arrest in T98G cells. In addition, high doses of methadone (20 µM) may delay G_1_/S transition, and thereby, cell cycle progression in unirradiated U251 cells.

We have previously shown that experimental interference with electrosignaling and cell cycle control decreases the clonogenic survival of irradiated tumor cells [95,99,100,101,102,103]. We, therefore, tested whether the observed methadone (20 µM)-mediated modulation of cell cycle control was associated with an impairment of clonogenic survival and radioresistance. 

**Figure 4:**
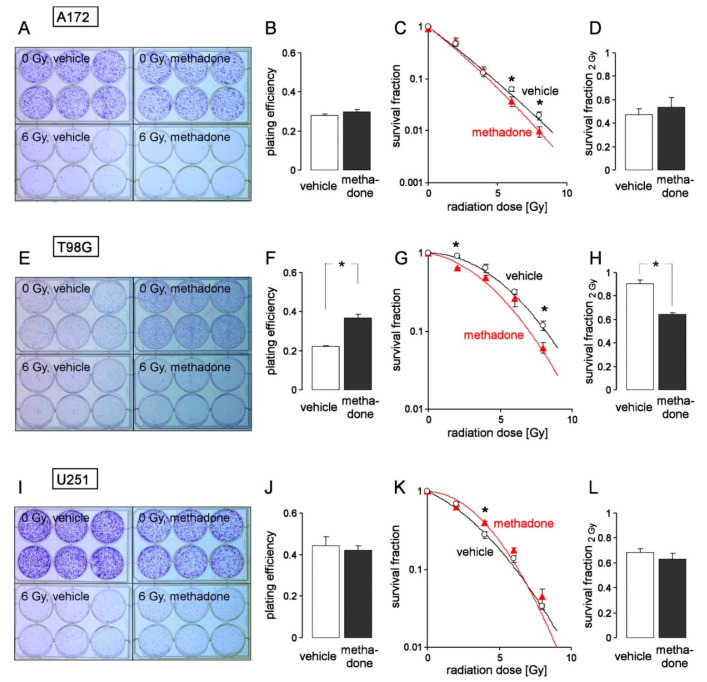
Heterogeneous effects of high-dose methadone (20 µM) on clonogenic survival of irradiated (0–8 Gy) human A172 (**A**–**D**), T98G (**E**–**H**) an U251 (**I**–**L**) glioblastoma cells. (**A**,**E**,**I**) Coomassie blue-stained colonies regrown from single 0 Gy (upper row)- and 6 Gy (lower row)-irradiated A172 (**A**), T98G (**E**), and U251 (**I**) glioblastoma cells pre- (1 h) and post-incubated (24 h) with either 0 μM (vehicle, ethanol, left) or 20 μM (right) methadone. (**B, F, J)** Mean (± SE, n = 11–12) plating efficiency of A172 (**B**), T98G (**F**), and U251 (**J**) glioblastoma cells incubated (24 h) with either 0 μM (vehicle, open bar) or 20 μM (closed bar) methadone. (**C**,**D**,**G**,**H**,**K**,**L**) Mean (± SE, n = 11–12) survival fraction of irradiated (0, 2, 4, 6, and 8 Gy in (**C**,**G**,**K**) and 2 Gy in (**D**,**H**,**L**)) A172 (**C**,**D**), T98G (**G**,**H**), and U251 (**K**,**L**) cells irradiated and pre- (1 h) as well as post-incubated (24 h) with either 0 μM (vehicle, open circles and open bars) or 20 μM (red triangles and closed bars) methadone. * indicates *p* ≤ 0.05, two-tailed (Welch-corrected) *t*-test. For delayed plating colony formation, cells were irradiated (0, 2, 4, 6, 8 Gy) and post-incubated for 24 h in the absence (vehicle) or presence of methadone (20 µM). Then, cells were replated in methadone-free medium and grown for a further two weeks. Thereafter, colonies were defined as cell clusters of ≥50 cells and colony numbers were counted. Plating efficiency (PE) was defined by the ratio between counted colonies (N) and plated cells (n) under control conditions (PE = N/n). The survival fraction (SF) was calculated by normalizing in both arms (methadone and vehicle control) separately the plating efficiency after irradiation (PE_xGy_) to that of the corresponding unirradiated control (PE_0Gy_) by the formula SF = PE_xGy_/PE_0Gy_. SFs were fitted by the use of the linear quadratic equation (SF = e^-(α·D + β·D^2)^ with D = radiation dose and α and β cell-specific constants).

To determine clonogenic survival of T98G, U251 and A172 glioblastoma cells by delayed plating colony formation assay, cells were pretreated for 1 h with a “supratherapeutic” concentration of methadone (20 μM) or vehicle alone (ethanol), irradiated with 0, 2, 4, 6 or 8 Gy and post-incubated (24 h) in the presence (20 μM) and absence (vehicle) of methadone, before washing and reseeding the cells for colony formation in the absence of the drug. As a result, methadone increased the plating efficiency of T98G cells but not of U251 and A172 cells (Figure 4A,E,I, upper row, and Figure 4B,F,J). In addition, methadone had little or contrary effects on survival fractions of the individual irradiated glioblastoma lines tested (Figure 4A,E,I, lower row, and Figure 4C,G,H). In A172 (Figure 4C) and T98G (Figure 4G) cells, methadone (20 µM) slightly radiosensitized the cells at some but not all of the applied radiation doses. In U251, in contrast, methadone promoted radioresistance in 4 Gy-irradiated cells. The survival fraction at 2 Gy (SF_2 Gy_), which is clinically relevant because 2 Gy are applied per daily fraction in normofractionated protocols, was reduced by methadone only in T98G cells (Figure 4D,H,L). Combined, the data indicate that a “supratherapeutic” concentration of methadone may have both clonogenic survival-promoting and impairing effects in control and irradiated glioblastoma cells. In particular, in T98G cells, the methadone-induced decrease in radioresistance (decline of SF_2 Gy_) was probably compensated by the methadone-stimulated clonogenicity (plating efficiency), suggesting that methadone even at a very high concentration (that is assumed to modulate several molecular target proteins beyond the µ-opioid receptor) does not exert clinically relevant beneficial effects alone or concomitant to radiotherapy.

**Figure 5:**
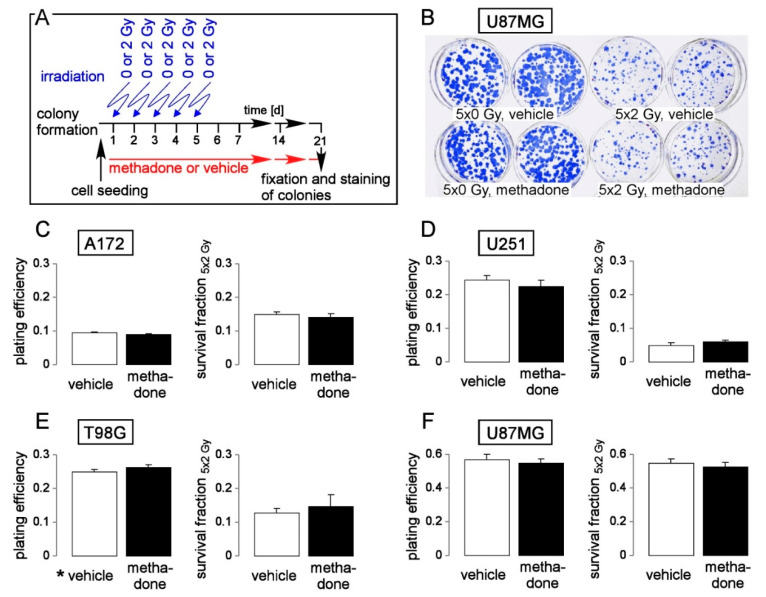
Methadone in a clinically relevant concentration (2 µM) does not impair clonogenic survival or radioresistance of human glioblastoma cells. (**A**) Experimental protocol of pre-plating colony formation depending on methadone concentration (0 or 2 µM) and fractionated irradiation (5 × 0 or 5 × 2 Gy). (**B**) Coomassie blue-stained colonies regrown from 5 × 0 Gy (**left**)- and 5 × 2 Gy (**right**) U87MG cells co-incubated with either 0 μM (vehicle, ethanol, upper row) or 2 μM (lower row) methadone. (**C**–**F**). Mean (± SE, n = 6) plating efficiency (**left**) and survival fraction 5 × 2 Gy (**right**) of A172 (**C**), T98G (**D**), U251 (**E**), and U87MG (**F**) glioblastoma cells co-incubated with either 0 μM (vehicle, open bars) or 2 μM methadone (closed bars). For pre-plating colony formation, plated cells were irradiated (0 or 2 Gy on days 1, 2, 3, 4, and 5) and post-incubated in the absence (vehicle, ethanol) or presence of methadone (2 µM) until formation of colonies. Thereafter, plating efficiencies and survival fractions were calculated as described in the legend of Figure 4.

Finally, we adjusted our protocol for analysis of clonogenic survival towards a clinically more relevant setting: T98G, A172, U251 and a further human glioblastoma cell line, U87MG, were plated and irradiated in five consecutive days with 0 or 2 Gy each and post-incubated for a further 2–3 weeks until formation of colonies. This pre-plating colony formation assay was carried out in the continuous absence (vehicle alone, ethanol) or presence of methadone (2 µM, Figure 5A). As a result, the clinically relevant methadone concentration of 2 µM did neither decrease plating efficiency (Figure 5B–F, left) nor the survival fraction (SF 5 × 2 Gy, Figure 5B–F, right) indicating that methadone in clinically achievable concentrations neither impairs clonogenicity nor radioresistance of glioblastoma cells in vitro.

## 6. Concluding Remarks

Recommended plasma methadone concentrations for patients in a methadone maintenance program are in the range of 0.5–2 µM (150–600 ng/mL), which are usually achieved by daily doses of up to 120 mg. Importantly, data from patients who died from methadone overdose suggest an overlap of therapeutic and toxic concentrations and, thus, a narrow therapeutic window. Therefore, methadone dose escalation strategies are not feasible. The µ-opioid receptor is not detected in the majority of glioblastoma specimens. Other molecular methadone targets such as NMDA receptors, in contrast, are frequently expressed in glioblastoma and are assumed to become modulated by therapeutic methadone concentrations. Nevertheless, preclinical data strongly suggest that methadone at clinically relevant concentrations in combination with radiotherapy or temozolomide chemotherapy, or temozolomide-radiochemotherapy does not exert any anti-glioblastoma effect. In addition, clinical data from patients under methadone maintenance therapy or glioblastoma patients with methadone prescriptions for other reasons, do also not suggest anyanti-glioblastoma activity of methadone. Therefore, patients need to be informed about the lack of evidence, and outside of clinical trials, the use of methadone to enhance anti-glioblastoma efficacy should be discouraged.

## Figures and Tables

**Table 1:** Clinically observed methadone tissue concentrations.

Application	Incidence	Daily Oral Dose [mg]	Organ	Methadone [µM]	Patient Number	Ref.
anti-glioma therapy	methadone maintenance	30	blood	0.59–0.67	1	[19]
drug substitution	methadone maintenance	83.3 *	blood	1.5 * 2 ^#^	104	[46]
drug abuse	methadone-associated death		Blood	2.6 * 14.5 ^#^	17	[38]
			brain	12.0 *^,$^	17	
drug substitution/drug abuse	methadone-associated death	20–80	Blood	1.6 ** 10.6 ^#^	15	[37]
			brain	2.2 **^,$^ 8.6 ^#,$^	15	
			CSF	0.7 ** 2.8 ^#^	8	
drug substitution/drug abuse	^§^ methadone-associated death		blood	2.6 * (2.3 **) 8.5 ^#^	52	[47]
drug substitution/drug abuse	^§^ methadone-associated death		blood	1.3 * (2.3 **) 9.7 ^#^	11	[48]

^$^ roughly estimated from ng/g data under the assumption of equal distribution between all intracellular and extracellular compartments; * mean; ** median; ^#^ highest value; ^§^ not in combination with other drugs; CSF: cerebrospinal fluid.

**Table 2:** Methadone sensitivity of proposed methadone target proteins.

Protein	Gene	Cell Model	Cellular Effects	Methadone IC_50_	Enantiomer	Refs
*N*-methyl-d-aspartate receptors	GRIN1 GRIN2A GRIN2B GRIN2C GRIN2D	Heterologous Xenopus laevis Expression system rat cortical membranes spinal cord rat forebrain spinal cord	blockage of NMDA currents	≥~2 µM ≥~0.1 µM ≥~4 µM ~3.1 µM >~3.1 µM ~18 µM ~4.3 µM	R(−)/S(+) R(−) S(+) R(−)/S(+) R(−)/S(+) R(−)/S(+) R(−)/S(+)	[63,64,65]
L type Ca^2+^ channels T type Ca^2+^ channels	CACNA1C CACNA1D CACNA1S CACNA1F CACNA1G CACNA1H CACNA1I	mouse neuroblastoma	Blockage of T- and L-type Ca^2+^ currents	>~10 µM	R(−)/S(+) ?	[66]
α3β4 nicotinic receptor	CHRNA3 CHRNB4	nAChRs-transfected HEK293 cells	blockage of 86Rb^+^ efflux	1.9 ± 0.2 μM	R(−)/S(+)	[67]
hERG1	KCNH2	GH_3_ pituitary tumor cells HEK293 stably expressing hERG	blockage of hERG currents	~10 µM 7 µM 2 µM	R(−)/S(+) R(−) S(+)	[68,69]
GIRK1/GIRK2	KCNJ3/KCNJ6	Xenopus laevis oocytes injected with mRNA	blockage of GIRK1/GIRK2 currents	~53 μM	R(−)/S(+)	[70]
Na_v_1.2 Na_v_1.3 Na_v_1.7 Na_v_1.8	SCN2A SCN3A SCN9A SCN10A	mouse peripheral nerves	blockage of excitability	86–119 µM	R(−)/S(+)	[71]
ClC-2	CLCN2	T84 intestinal cells HEK293EBNA stably expressing hClC-2	blockage of hClC-2 currents	100 Nm 100–230 nM	R(−)/S(+) R(−)/S(+)	[72]
p-glyco-protein (hMDR)	ABCB1	hMDR -transfected HEK293 cells human placental inside-out vesicles	calcein efflux paclitaxel uptake	>25 µM K_i_ = 18 µM	R(−)/S(+) R(−), S(+) R(−)/S(+)	[73,74]

Methadone is well known as a non-competitive antagonist for the *N*-methyl-d-aspartate (NMDA) receptor. In various studies, IC_50_ values for methadone between ~0.1 and ~18 µM were measured [63,64,65] with conflicting data on stereospecificity towards R(-)-(L-)- and S(+)-(D-)-enantiomers [63,65]. ?: not detailed in the quoted original article which enantiomer was used.

**Table 3:** Tumoricidal methadone effects in preclinical studies as monotherapy or in combination with other anti-tumor therapies.

Tumor Model Carcinoma Cells	Methadone	Read-Out	Effects	Ref
H187 (SCLC) and H157 (NSCLC) cells	0.01–0.1 µM	dehydroge- nase activity, trypan blue exclusion, delayed plating growth assay	impaired viability IC_50_ 0.3–10 nM	[14]
NCI-N417 (SCLC), NCI-H460 (NSCLC) *xeno*grafts	10 mg/(kg d)	tumor volume	growth delay	[14]
MIA PaCa-2 pancreatic and HT-29 colon adeno-carcinoma, CAL-27 (HNSCC) cells	10 µM	TUNEL, annexin-V binding, trypan blue exclusion	cell viability was not altered	[86]
FaDu, HLaC78 and PJ41 (HNSCC) cells	2–32 µM	dehydroge- nase activity	impaired viability IC_50_ >> 32 µM cisplatin-, doxorubicin-, 5-FU- and paclitaxel sensitization IC_50_ ≥ 32 µM	[84]
FaDu (HNSCC) cells	32 µM	annexin-V binding	cell viability was not altered, enhancement of ALA-PDT	[87]
T24 and HT-1376 bladder cancer cells	0.3–32 µM	dehydroge- nase activity, annexin-V binding PI staining	impaired viability IC_50_ >> 32 µM cisplatin sensitization ~32 µM and >>32 µM	[85]
**leukemia**				
CEM and HL-60 leukemia cells	10–30 µM	subG_1_ population PI-staining	apoptotic cell death IC_50_ ~15 µM	[17]
CCRF-CEM and HL-60 leukemia cells	60–200 µM	dehydroge-nase activity	impaired viability IC_50_ ≥ 100 µM	[78]
ALL leukemia cells	1–323 µM	cell number	impaired viability IC_50_ >20 µM	[79]
ALL leukemia cells	0.3–32 µM	subG_1_ population PI-staining	apoptotic cell death, IC_50_ >32 µM, doxorubicin sensitization IC_50_ <0.3–≥32 µM	[18]
ALL leukemia cells	20 µM	western blot, cell viability assay	OPMR1 knockdown enhanced asparaginase resistance, methadone sensitized to asparaginase treatment	[80]
ALL leukemia *xeno*grafts	20–120 mg/(kg d)	tumor volume	growth delay, doxorubicin sensitization	[18]
**neuroblastoma**				
SH-SY5Y human neuroblastomacell line	100–1000 µM	LDH activity, caspase activity, cyt-c release, ATP concentration	caspase independent cell death, bioenergetic crisis IC_50_ ~500µM,	[83]
**glioblastoma**				
U118MG and A172 glioblastoma cells	3.2–32 µM	subG_1_ population PI-staining	cell death IC_50_ > 32 µM, doxorubicin sensitization IC_50_ ≤3 - ~10 µM	[16]
U87MG glioblastoma *xeno*grafts	60–120 mg/(kg d)	tumor volume	growth delay	[16]
U87MG, U251 and primary glioblastoma cells	1–145 µM	crystal violet staining, annexin-V- - binding	impaired viability/apoptotic cell death IC_50_ (25) ≥100 µM, TMZ sensitization IC_50_ (~50) >>145 µM	[81]
primary glioblastoma cells	1–30 µM	ATP concentration, dehydroge- nase activity	impaired viability IC_50_ between 10 and 30 µM, TMZ sensitization IC_50_ >> 30 µM, radiosensitization IC_50_ >> 30 µM	[91]
A172 glioblastoma cells	32 µM	annexin-V binding, 7- 7-AAD exclusion	unaltered viability, enhancement of ALA-PDT	[87]
A172 glioblastoma cells	0.065 µM	annexin-V binding	apoptotic cell death, enhancement of ALA-PDT	[82]
A172 glioblastoma cells	2–32 µM	dehydroge- nase activity	impaired viability IC_50_ >>32 µM cisplatin-, doxorubicin-, 5-FU- and paclitaxel sensitization IC_50_ ≤4 - ≥32 µM	[84]

SCLC: small cell lung cancer, NSCLC: non-small cell lung cancer, HNSSC: head and neck squamous cell carcinoma, PI: propidium iodide, 7-AAD: 7-amino-actinomycin D, ALA-PDT: 5-aminolevulinic acid-based photodynamic therapy, 5-FU: 5-fluoruracil.
